# Comparison Effect of Intravenous Ketamine with Pethidine for Analgesia and Sedation during Bone Marrow Procedures in Oncologic Children: A Randomized, Double-Blinded, Crossover Trial

**Published:** 2016-10-01

**Authors:** Babak Abdolkarimi, Soheila Zareifar, Majid Golestani Eraghi, Fazl Saleh

**Affiliations:** 1 Assistant Professor of Hematology-Oncology, Department of Pediatrics, Lorestan University of Medical Sciences, Khoramabad, Iran; 2 Associate Professor of Hematology-Oncology, Department of Pediatrics, Amir Oncology Hospital, Shiraz University of Medical Sciences, Shiraz, Iran; 3 Fellowship in Intensive Care, Anesthesiologist, Tracheal Diseases Research Center, National Research Institute of Tuberculosis and Lung Diseases (NRITLD), Shahid Beheshti University of Medical Sciences, Tehran, Iran; 4 Fellowship in Intensive Care, Anesthesiologist, Lung Transplantation Research Center, National Research Institute of Tuberculosis and Lung Diseases (NRITLD), Shahid Beheshti University of Medical Sciences, Tehran, Iran; 5 Fellowship of Hematology-Oncology Department of Pediatrics, Amir Oncology Hospital, Shiraz University of Medical Sciences, Shiraz, Iran

**Keywords:** Cancer, Pain, Pediatric, Analgesia

## Abstract

**Background** **:** Children suffering from cancer always require pain relief and reduce anxiety when undergoing painful procedures. The aim of this study is to compare the effect of pethedine and ketamine administration in cancer-diagnosed children undergoing bone marrow aspiration and biopsy procedures.

**Subjects and Methods** **:** A randomized, double-blinded, crossover trial was carried out on 57 children undergoing painful procedures (bone marrow aspiration/biopsy). Patients were randomly assigned in a double-blinded fashion to receive either intravenous pethedine (1 mg/kg/dose) or ketamine (1 mg/kg/dose), respectively. The effectiveness of the drug was measured utilizing three parameters; perception of procedural pain with Wong-Baker Faces Pain Rating Scale and Richmond Agitation-Sedation Scale (RASS), hemodynamic changes and respiration and the frequency of vomiting nausea score.

**Results** **:** Additionally, hemodynamic stability and pain control were significantly better in the patients receiving ketamine (p<0.05, at 0, 15, 30 min). Nausea and vomiting were more frequent in Group K than in Group M but there were no significant differences. No serious complications were observed.

**Conclusion:** This study showed that intravenous ketamine generated a superior clinical effect in decreased pain. Ketamine may also be recommended as a reasonable option before oncology procedures in children suffering from cancer.

## Introduction

 Bone marrow aspiration and biopsy are trouble and painful procedures for pediatric patients. Performing this procedure with minimum pain and mental sequel is an ideal target for pediatric oncologists.^1^^,^^2^ Some drugs such as narcotics were administered for performing analgesia during this procedure but there are a few experience in children as a result of the adverse effects. Physicians are familiar with the side effects of these medications, the one that raises more concern is when patient's breathing is reduce or stop, as well as dangerously lowering their blood pressure. An alternative medication is ketamine. This medication is also commonly utilized in the emergency department; however, it is typically used to help sedate patients for uncomfortable procedures. Ketamine has also been utilized for pain control. Therefore, administration of safe alternative drug instead of morphine or pethidine is a critical point for short term analgesia for pediatrics. Ketamine was not administered as a short term analgesic agent for children.^3^^-^^6^

**Figure 1:**
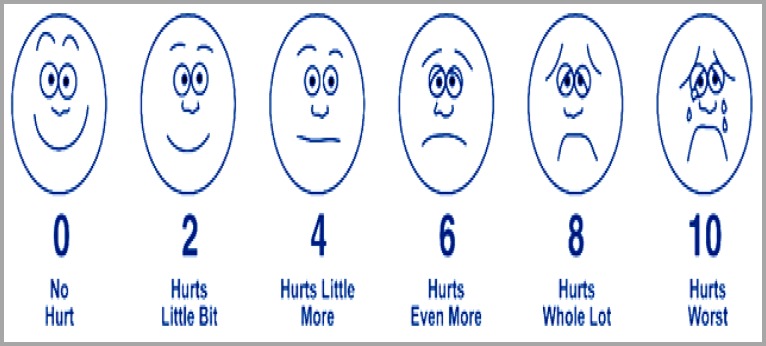
Wong-Baker Faces Pain Rating Scale

The Wong-Baker Faces Pain Rating Scale and Richmond Agitation-Sedation Scale (RASS) are tools for the determination of pain scoring in children. The Wong-Baker Faces Pain Rating Scale (Figure 1) is a pain scale that shows a series of faces ranging from a happy face at 0, "No hurt" to a crying face at 10 "Hurts worst". The patient must choose the face that best describes how they are feeling.^7^

The Richmond Agitation-Sedation Scale (RASS) (Table 1) is a ten point scale with levels for assessing anxiety and agitation, one for an alert and calm state with further levels for quality of sedation. RASS can be assessed in 30-60s and does not require equipment.^8^^,^^9^ Three sequential steps are used: observation, response to verbal stimulation and response to physical stimulation. A unique feature of RASS is that it uses the duration of eye contact following verbal stimulation as the principal means of titrating sedation.^10^

## SUBJECTS AND METHODS

 In this prospective, randomized, double-blind clinical study was carried out between December 2012 and February 2014, 57 children within the age group of 5 to 15 years posted for elective surgical procedures were enrolled.

**Table 1:** Richmond Agitation-Sedation Scale

**Score**	**Term**	**Description**
**+4**	Combative	Overtly combative or violent; immediate danger to staff
**+3**	Very agitated	Pulls on or removes tube (s) or catheter (s) or has aggressive behavior toward staff
**+2**	Agitated	Frequent, non purposeful movement or patient, ventilator dyssynchrony
**+1**	Restless	Anxious or apprehensive but movements not aggressive or vigorous
**0**	Alert and calm	Spontaneously pays attention to caregiver
**-1**	Drowsy	Not fully alert, but has sustained (more than 10 seconds) awakening, with eye contact, to voice
**-2**	Light sedation	Briefly (less than 10 seconds) awakens with eye contact to voice
**-3**	Moderate sedation	Any movement (but no eye contact) to voice
**-4**	Deep sedation	No response to voice, but any movement to physical stimulation
**-5**	Unarousable	No response to voice or physical stimulation

Ethics committee approval was obtained by Research Advisory Council (RAC) at Shiraz University of Medical Sciences. Informed written consents were obtained from the parents of participants. All patients were examined preoperatively by similar anesthesiologist and oncologist. Children with history of allergy to any of drugs used in this study as well as children receiving anticonvulsants, sedatives or analgesics in the preoperative period were excluded from the study. Patients with mediastinal mass were also excluded from the study. The sample size (60 people) was calculated (α=0.05 and d=0. 12).

Randomization was achieved using randomly permuted blocks and software "research randomizer" at: http://www.randomizer.org.

Those included in study were randomly allocated to one of the two groups: 'Group K' and 'Group P'. Group K received combination of intravenous (IV) ketamine (1 mg/kg) and midazolam (0.1 mg/kg) while Group P received IV midazolam (0.1 mg/kg) and pethidine (1 mg/kg). Every group also received same dose of IV atropine (0.04 mg/kg) to avoid hyper secretion due to ketamine. The following parameters were assessed (include mean arterial pressure, respiratory rate, RASS score, Wong-Backer score, drug adverse effects) before drugs injection and at 10 (at beginning of procedure) and 30 min after administering premedication. The heart and respiratory rates and mean arterial pressure (MAP) were monitored during procedures. Sedation was scored using a five point scale (RASS scoring) including agitated (clinging to parents or crying), awake (alert but not clinging to the parents, may whimper but not cry, anxious), sleeping intermittently (relaxed, less responsive), asleep (response to minor stimulation e.g. light touch, soft voice), barely arousable (arousable by persistent stimulation needs shaking or shouting to arouse).

The intensity of pain was also assessed using Wong-Backer score before the procedure was carried out.

**Statistical analysis**

Continuous data with normal distribution are given as mean ± standard deviation; otherwise, as median, independent t-test for testing the significance of mean for independent continuous scale (of normal distribution) data, Mann-Whitney for testing the significance of mean for non-normal distribution data, Chi-squared or Fisher exact test for testing the significance of percentages (qualitative data) were used as the statistical tools.

Hemodynamic changes during the procedure were compared between groups by repeated measure of ANOVA (for normal distribution); Friedman non-parametric test was utilized for the significance of non-normal distribution data approach with treatment group and time as the between- and within-group factors.

χ^2^ (Chi-squared) tests. Arterial blood pressures and respiratory rates were compared using Student’s t-test. The effects of intravenous administration of pethidine analgesia and intravenous ketamine analgesia were assessed by repeated measures analysis of variance (RM-ANOVA).

Statistical analyses were carried out using the statistical package for social sciences, version 16.0 (SPSS, Chicago, Illinois). A p-value <0.05 was considered statistically significant.

## Results

 Results were presented for 57 patients. Sixty patients were enrolled in this study; three subjects were eliminated from data analysis due to the following reasons: Two refused to participate after enrolling while one patient left the hospital before the bone marrow procedure. The patients were divided into two groups: Group K which includes 27 patients who received ketamine-midazolam while Group P includes 30 patients who received pethidine-midazolam (p>0.05) (Table 2).

**Table 2:** Variable characteristics of Ketamine and pethidine groups in terms of sedation schedule

**Measured variable**		**Pethidine ** **group (n=30)**	**Ketamine ** **group (n=27)**	**p-value**
**Heart rate/min**	0 10 30	87.6 ± 11.17 79.78 ± 11.63 76.58 ± 1035	84.83 ± 7.52 76.45 ± 5.69 72.09 ± 4.30	p-value >0.05
**Respiratory ** **rate/min**	0 10 30	16.3 ± 3.9 15.15 ± 3.05 14.0 ± 2.2	17.15 ± 2.95, 15.2 ± 3.1 19.1 ± 2.8	p-value >0.05
**Mean arterial ** **pressure ** **(mmHg)** **/min**	0 10 30	96.78 ± 6.27 91.53 ± 5.39 94.58 ± 7.35	93.48 ± 8.53 89.83 ± 4.36 86.00 ± 4.21	p-value < 0.05
**Oxygen ** **saturation ** **(%)/min**	0 10 30	98.7 98.8 98.8	98.7 98.6 98.6	p-value >0.05

RASS score was comparable between the groups with median score of +4 in both groups. RASS score at 0, 15, 30 min after sedation was not comparable between the groups (Table 3). Rate of nausea and vomiting did not have statistically significant differences between patients in two groups (p=0.6150, p=0.576). Pruritus was not seen in the two groups. There were no significant differences between the groups in terms of demographic characteristics such as age, sex and weight (Table 4).

**Table 3:** Variable characteristics of ketamine and pethidine groups in Wong and RASS Score

**Measured variable**		**Pethidine ** **group ** **(n=30)**	**Ketamine ** **group ** **(n=27)**	**p-value**
**Wong ** **score**	0 min 10 min 30 min	6 (0-7) 7 (1-10) 4 (1-10)	6 (2-10) 3 (0-10) 2 (2-8)	p-value<0.05
**mean of ** **RASS ** **score**	0 min 10 min 30 min	1.5 ± 0.50 1.8 ± 0.50 1.8 ± 0.40	1.7 ± 0.6 2.1 ± 0.4 2.1 ± 0.4	p-value=0.45 p-value=0.32 p-value=0.32

**Table 4:** Variables characteristics of ketamine and pethidine groups in demographic variables

**Measured Variable**		**Pethidine ** **group ** **(n=30)**	**Ketamine ** **group ** **(n=27)**	**p-value**
**Sex**	Female 33 (58%) Male 24 (42%)	13 (23%) 19 (33%)	20 (35%) 5 (9%)	p-value >0.05
**Age (year)**		12.4 ± 2.93	10.9 ± 3.05	p-value >0.05
**Weigh (kg)**		23.2 ± 8.89	9.9 ± 25.46	p=0.569

## Discussion

 Deep sedation for painful procedure in children is often problematic. Sedative-analgesic drugs and ways for painful procedures in oncology ward have been less studied when compared to other wards. Children with cancer may remember the bad memory due to the painful procedure, especially the frequent order at which these trouble experiences occur.

Our study evaluated and compared the efficacy of low dose intravenous midazolam combined with ketamine or pethidine in child patients. We did not use placebo group because bone marrow procedure is very painful without the administration of analgesia. After administration of oral ketamine, sedation occurred within 15–20 min which is similar to other oral premedication regimens. The bioavailability of oral ketamine and oral midazolam are 10-16% and 40-50%, respectively due to extensive first pass hepatic extraction^11^, but we used premedication drugs in intravenous form with low effective dose of two drugs for the prevention of first pass hepatic effect. In comparison of the efficacy of oral ketamine (10 mg/kg) to intramuscular morphine (0.1 mg/kg), both in combination with trimeperazine (3 mg/kg), as anesthetic premedicant during pediatric cardiac surgery, there are no significant differences in patient arousal or cooperation with induction of anesthesia were found. No adverse effects of ketamine were observed.^12^^-^^14^

Although our study showed that there were no statistically significant differences in heart and respiratory rates between the two groups over time and it was clinically insignificant, mean arterial pressure difference was significant. There was an increase in heart rate in both groups from baseline and it might not be due to the use of atropine since its action starts within 30 min and peaks at one hour. There was no statistically significant change in cardiorespiratory variables between groups as time progresses.

No harmful effect of ketamine and midazolam on cardiorespiratory system was confirmed.^15^^-^^17^ Also, ketamine has no important circulatory, respiratory or neurological side effects which were attributable to either premedication.^18^^,^^19^

In pediatric oncology ward using propofol and midazolam with fentanyl orondansetron for children undergoing bone marrow aspiration and intrathecal chemotherapy could reduce pain but we did not find any study on ketamine for children undergoing bone marrow procedure.^20^^-^^22^ In comparison of midazolam/fentanyl versus midazolam/ketamine administered through central venous catheter in pediatric intensive care unit (PICU) and found that the group that received midazolam/ketamine was noted to have more minor complications such as hypersecretion, desaturation, aspiration and temporary airway obstruction.^22^^-^^24^ The generalization of these findings to younger children and across all cultures may not be applicable. However, it is unlikely that these factors would have caused any change in our results, since the study population was school-aged children and adolescents. Fear and anxiety may result in bias when reporting pain and interfere with attempts at measuring pain intensity.

**Limitations of study**

This was a single-center study in which patients were enrolled as a convenience sample according to predetermined inclusion and exclusion criteria. We also did not assess the impact of these drugs on recovery from anesthesia.

## CONCLUSION

 Combination of ketamine and midazolam has a better analgesic effect and lesser hemodynamic changes during sedation than combined pethidine and midazolam in pediatric patients undergoing bone marrow procedure. The combination of low cost, high efficacy and apparent safety made fentanyl an attractive option to be used as premedication for the older patients undergoing bone marrow procedures and intrathecal chemotherapy.
